# Uric Acid Level Has a U-Shaped Association with Loss of Kidney Function in Healthy People: A Prospective Cohort Study

**DOI:** 10.1371/journal.pone.0118031

**Published:** 2015-02-06

**Authors:** Eiichiro Kanda, Toshitaka Muneyuki, Yoshihiko Kanno, Kaname Suwa, Kei Nakajima

**Affiliations:** 1 Department of Nephrology, Tokyo Kyosai Hospital, Meguro, Tokyo, Japan; 2 Center for life science and bioethics, Tokyo Medical and Dental University, Bunkyo, Tokyo, Japan; 3 Saitama Citizens Medical Center, Saitama, Saitama, Japan; 4 Department of Nephrology, Tokyo Medical University, Shinjuku, Tokyo, Japan; 5 Saitama Health Promotion Corporation, Hikigun, Saitama, Japan; 6 Division of Clinical Nutrition, Department of Medical Dietetics, Faculty of Pharmaceutical Sciences, Josai University, Sakado, Saitama, Japan; University of São Paulo School of Medicine, BRAZIL

## Abstract

**Background:**

The relationship between hyperuricemia and chronic kidney disease (CKD) has been found in various observational studies. Although hypouricemia is associated with cardiovascular events, it has not been established as a risk factor for CKD. We investigated the relationship between serum uric acid level and the loss of kidney function and incident CKD in healthy people.

**Materials and Methods:**

Healthy people were enrolled in this community-based prospective cohort study, the Saitama Cardiometabolic Disease and Organ Impairment Study, Japan. The analysis was conducted on 4188 subjects followed up for at least 3 years, 3102 for 6 years and 1052 for 9 years. Their data including glomerular filtration rate (eGFR) decline were examined every three years. The outcome event was incident CKD or the decrease in eGFR by more than 25% in three years. Multivariate statistical models were adjusted for the baseline characteristics.

**Results:**

The following data was obtained: mean±SD age, male, 39.6±10.4 years, female 38.4±10.8 years; eGFR, male, 81.9±16.4 ml/min/1.73m2, female, 82.1±17.5 ml/min/1.73m2; serum uric acid level, male, 5.8±1.2 mg/dl, female, 4.1±0.9 mg/dl. Both low and high serum uric acid levels were associated with the outcome and eGFR decline in males (multivariate logistic additional additive models, linear *p* = 0.0001, spline *p* = 0.043; generalized additive models, linear *p* = 0.0001, spline *p* = 0.012). In subjects with low serum uric acid levels (male, <5 mg/dl; female, <3.6 mg/dl), multivariate linear mixed models showed that low serum uric acid levels were associated with eGFR decline in a time-dependent manner (male, *p* = 0.0001; female, *p* = 0.045).

**Conclusion:**

This study showed that low as well as high levels of uric acid are associated with the loss of kidney function. Hypouricemia is a candidate predictor of kidney function decline in healthy people.

## Introduction

Hyperuricemia is frequently observed in patients with chronic kidney disease (CKD) and has been reported as a risk factor for the progression and development of CKD [1–4]. Serum uric acid level has also been reported to be associated with cardiovascular death. A U-shaped association between serum uric acid level and cardiovascular mortality has been reported, suggesting that both hyperuricemia and hypouricemia are risk factors for cardiovascular death [5,6]. A U-shaped association between serum uric acid level and the loss of kidney function has not been reported thus far to the best of our knowledge.

Patients with hypouricemia accounted for approximately 0.5% of the normal population [7]. Hypouricemia is caused by suppressed renal tubular reabsorption and uric acid production; the former being more frequently reported [8–10]. Patients with renal hypouricemia tend to develop acute kidney injury (AKI) after a strenuous exercise [11]. However, the risk of CKD development in patients with hypouricemia has not yet been clarified.

The Saitama Cardiometabolic Disease and Organ Impairment Study (SCDOIS) in Japan is a community-based prospective cohort study [12,13]. Through this study, community-based data from medical checkups of asymptomatic people have been obtained. We examined the association between serum uric acid level and the loss of kidney function using the longitudinal SCDOIS data.

## Materials and Methods

### Data Source

SCDOIS was a multidisciplinary observational epidemiological research study conducted in Saitama prefecture, Japan [12,13]. In brief, this study started in 2011 and involved three institutions in Saitama, namely, Josai University, Jichi University, and Saitama Health Promotion Corporation. The protocol of this study was in accordance with the Declaration of Helsinki, and was approved by the ethics committees of Josai University, Jichi University, and Saitama Health Promotion Corporation. Written informed consent regarding participation to this study was obtained at the time of the checkup from all the subjects. However, because the informed consent from the subjects was not obtained concerning the availability of their data to the public, there were restrictions on sharing of data.

**Fig 1:**
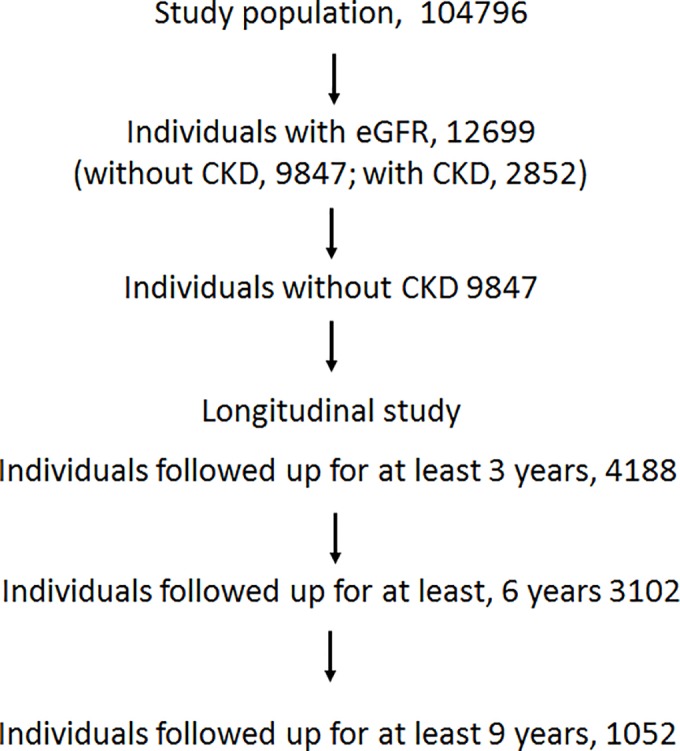
Flow diagram of subject classification. Subjects were followed up for 3 to 9 years. The analysis was conducted on 4188 subjects followed up for at 3 years, 3102 for at least 6 years, and 1052 for 9 years.

In this study, we analyzed data collected every three years from records of the medical checkups of asymptomatic people living or working in Saitama from 1999 to 2008. The study population consisted of 104796 subjects (Fig. 1). Individuals with missing data such as age, gender, and serum creatinine level were excluded from this study. Individuals with estimated glomerular filtration rate (eGFR) data (12699) were included in the study. Among 9847 individuals without CKD, the following were included as subjects in this study: 4188 followed up for at least 3 years; 3102 for 6 years; and 1052 for 9 years.

Baseline patient data, including age, gender, body mass index (BMI), comorbid conditions including diabetes mellitus (DM) and hypertension; histories of hyperuricemia including gout and cardiovascular diseases (CVDs); serum creatinine and uric acid levels; proteinuria; and habits of alcohol consumption, smoking, and regular exercise were collected from all the subjects. Data on age, BMI, serum uric acid level, eGFR, and proteinuria were collected every three years, and treated as time-dependent variables. eGFR was calculated using the following equation for the Japanese population [14]: eGFR (ml/min/1.73m^2^) = 194×serum Cr^-1.094^×age^-0.287^ (for female) ×0.739, where Cr = serum creatinine level (mg/dl). Annual GFR decline was calculated using the following formula: eGFR decline = (eGFR_after_ − eGFR_before_)/3.

### Statistical Analyses

Statistical analyses were carried out separately by gender. Data are presented as mean±SD. Intergroup comparisons were performed using the chi-square test, t-test, and one-way analysis of variance as appropriate. Multivariate statistical models were adjusted for the baseline characteristics, such as age, gender, BMI, comorbid conditions of hypertension and DM; histories of hyperuricemia including gout and CVDs; proteinuria; and habits of alcohol consumption, smoking, and regular exercise. The subjects were classified into four groups according to the quartiles of their serum urine levels. An outcome event was defined as incident CKD or a decrease in eGFR by more than 25%. Logistic additive models with spline adjusted for the baseline characteristics were used to examine the relationship between serum uric acid level and the likelihood of an outcome event. To examine the relationship between eGFR decline and serum uric acid level, multivariate linear mixed models were used for the repeatedly measured data, such as age, BMI, serum uric acid level, eGFR, and proteinuria. The effect of baseline serum uric acid level on eGFR decline was examined by generalized additive models with spline adjusted for the baseline characteristics. The effects of exercise load on the risk of incident CKD were examined. Exercise was defined as a more than 30-minute exercise with sweating. The exercise load categories were as follows: Males: level 1, almost no exercise; level 2, once a month; level 3, twice a month; level 4, once a week or more: Females: level 1, almost no exercise; level 2, once a month; level 3, twice a month or more. In females, the exercise load categories, twice a month and once a week or more, were treated as level 3, because the number of females who exercised once a week or more was small. Statistical analyses were performed using SAS version 9.2 (SAS Institute, Cary, NC). Values of *p* < 0.05 were considered statistically significant.

## Results

Subjects were categorized into four groups according to the quartiles of their serum uric acid levels. Their demographics including biochemical data are shown in Table 1 and 2. In males, group 4 (serum uric acid level, 6.5 mg/dl or higher) showed a higher BMI, a lower eGFR, and larger numbers of subjects with proteinuria, a history of hyperuricemia, and the habits of alcohol consumption than group 1 (serum uric acid level, lower than 5 mg/dl) (Table 1). In females, group 4 (serum uric acid level, 4.7 mg/dl or higher) showed a higher BMI, a lower eGFR, and a larger number of subjects with hypertension than group 1 (serum uric acid level, lower than 3.6 mg/dl) (Table 2).

**Table 1:** Baseline characteristics by gender-specific quartiles of serum uric acid levels (Male).

	All	Group 1	Group 2	Group 3	Group 4	p
N (%)	3148	710 (22.5)	757 (24.0)	850 (27.0)	831 (26.4)	
Age (years)	39.6±10.4	41.2±11.0	39.3±10.5	39.4±10.2	38.7±9.8	0.0001
BMI (kg/m^2^)	23.5±3.2	22.5±3.0	23.0±3.0	23.7±3.0	24.6±3.3	0.0001
Uric acid (mg/dl)	5.8±1.2	4.2±0.6	5.3±0.2	6.0±0.2	7.2±0.7	0.0001
eGFR (ml/min/1.73m^2^)	81.9±16.4	84.2±17.7	82.9±16.7	81.3±15.8	79.5±15.2	0.0001
Proteinuria (+) (%)	81 (2.6)	13 (1.8)	12 (1.6)	25 (2.9)	31 (3.7)	0.025
DM (%)	90 (2.9)	30 (4.2)	26 (3.4)	19 (2.2)	15 (1.8)	0.017
Hypertension (%)	153 (4.9)	42 (5.9)	27 (3.6)	39 (4.6)	45 (5.4)	0.16
Hyperuricemia (%)	95 (3)	11 (1.5)	6 (0.8)	10 (1.2)	68 (8.2)	0.0001
CVD (%)	62 (2)	9 (1.3)	22 (2.9)	17 (2)	14 (1.7)	0.13
Alcohol (%)	1918 (60.9)	391 (55.1)	426 (56.3)	528 (62.1)	573 (69)	0.0001
Smoking (%)	1474 (46.8)	327 (46.1)	348 (46)	424 (49.9)	375 (45.1)	0.20
Exercise (%)	1686 (53.6)	362 (51)	399 (52.7)	469 (55.2)	456 (54.9)	0.60
Incident CKD (%)	728 (23.1)	141 (19.9)	161 (21.3)	200 (23.5)	226 (27.2)	0.0036
More than 25% decrease in eGFR (%)	1248 (39.6)	310 (43.7)	308 (40.7)	340 (40)	290 (34.9)	0.0046
Outcome events (%)	1491 (47.4)	353 (49.7)	356 (47)	400 (47.1)	382 (46)	0.51
(%)	(47.4)	(49.7)		(47.1)		

Group 1, serum uric acid level was less than 5 mg/dl; group 2, 5 to less than 5.7 mg/dl; group3, 5.7 to less than 6.5 mg/dl; group 4, 6.5 mg/dl or higher.

Abbreviations: BMI, body mass index; eGFR, estimated glomerular filtration rate; DM, diabetes mellitus; hyperuricemia, history of hyperuricemia including gout; CVD, history of cardiovascular disease; alcohol, daily alcohol consumption; exercise, having regular exercise; CKD, chronic kidney disease; outcome event, incident CKD or more than 25% decrease in eGFR.

**Table 2:** Baseline characteristics by gender-specific quartiles of serum uric acid levels (Female).

	All	Group 1	Group 2	Group 3	Group 4	p
N (%)	1040 (100)	221 (21.3)	296 (28.5)	258 (24.8)	265 (25.5)	
Age (years)	38.4±10.8	37.5±9.6	38.1±10.1	38.2±11.2	39.6±11.8	0.17
BMI (kg/m^2^)	21.7±3.2	21.0±2.5	21.0±2.6	21.8±3.0	23.1±3.9	0.0001
Uric acid (mg/dl)	4.1±0.9	3.0±0.4	3.7±0.2	4.3±0.2	5.2±0.6	0.0001
eGFR (ml/min/1.73m^2^)	82.1±17.5	84.5±20.5	82.0±17.9	80.8±16.5	81.1±16.1	0.045
Proteinuria (+) (%)	21 (2)	4 (1.8)	8 (2.7)	3 (1.2)	6 (2.3)	0.62
DM (%)	10 (1)	2 (0.9)	1 (0.3)	4 (1.6)	3 (1.1)	0.52
Hypertension (%)	38 (3.7)	4 (1.8)	4 (1.4)	12 (4.7)	18 (6.8)	0.0021
Hyperuricemia (%)	2 (0.2)	0 (0)	0 (0)	0 (0)	2 (0.8)	0.12
CVD (%)	17 (1.6)	4 (1.8)	3 (1)	6 (2.3)	4 (1.5)	0.67
Alcohol (%)	226 (21.7)	42 (19)	61 (20.6)	60 (23.3)	63 (23.8)	0.42
Smoking (%)	92 (8.8)	18 (8.1)	22 (7.4)	30 (11.6)	22 (8.3)	0.33
Exercise (%)	372 (35.8)	70 (31.7)	106 (35.8)	96 (37.2)	100 (37.7)	0.47
Incident CKD (%)	349 (33.6)	65 (29.4)	96 (32.4)	87 (33.7)	101 (38.1)	0.23
More than 25% decrease in eGFR (%)	506 (48.7)	105 (47.5)	139 (47)	120 (46.5)	142 (53.6)	0.32
Outcome events (%)	581 (55.9)	123 (55.7)	166 (56.1)	134 (51.9)	158 (59.6)	0.37

Group 1, less than 3.6 mg/dl; group 2, 3.6 to less than 4.1 mg/dl; group 3, 4.1 to less than 4.7 mg/dl; group 4, 4.7 mg/dl or higher.

Abbreviations: BMI, body mass index; eGFR, estimated glomerular filtration rate; DM, diabetes mellitus; hyperuricemia, history of hyperuricemia including gout; CVD, history of cardiovascular disease; alcohol, daily alcohol consumption; exercise, having regular exercise; CKD, chronic kidney disease; outcome event, incident CKD or more than 25% decrease in eGFR.

### Incidence of Loss of Kidney Function and Serum Uric Acid Levels

**Fig 2:**
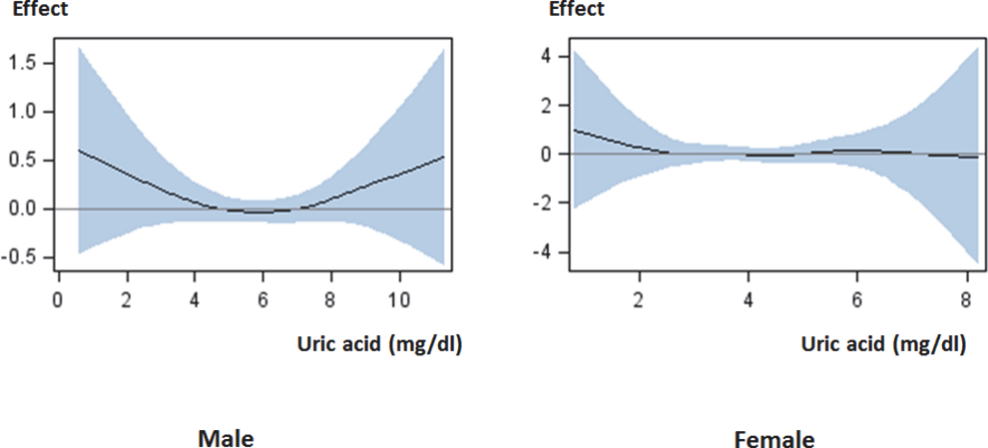
Effect of serum uric acid level on loss of kidney function. The high risk of loss of kidney function was observed in males with low or high serum uric acid levels, but not in females. The curves from logistic additive models with spline show a pattern indicating the effect of serum uric acid level on the likelihood of loss of kidney function with 95% confidence interval: male, linear *p* = 0.0001, spline *p* = 0.043; female, linear *p* = 0.18, spline *p* = 0.68. The models are adjusted for baseline characteristics. Abbreviations: Effect, effect of serum uric acid level on the likelihood of loss of kidney function.

A large number of males with incident CKD and more than 25% decrease in eGFR were observed in groups 4 and 1, respectively (Table 1). Moreover, there was no significant difference in the incidence of outcome events. In females, there was also no significant difference in the incidence of outcome events (Table 2). Logistic additive models showed that both high and low serum uric acid levels affected the likelihood of the outcome events than intermediate levels in males, and that serum uric acid level was not associated with the outcome events in females (Fig. 2).

### eGFR Decline and Serum Uric Acid Levels

**Fig 3:**
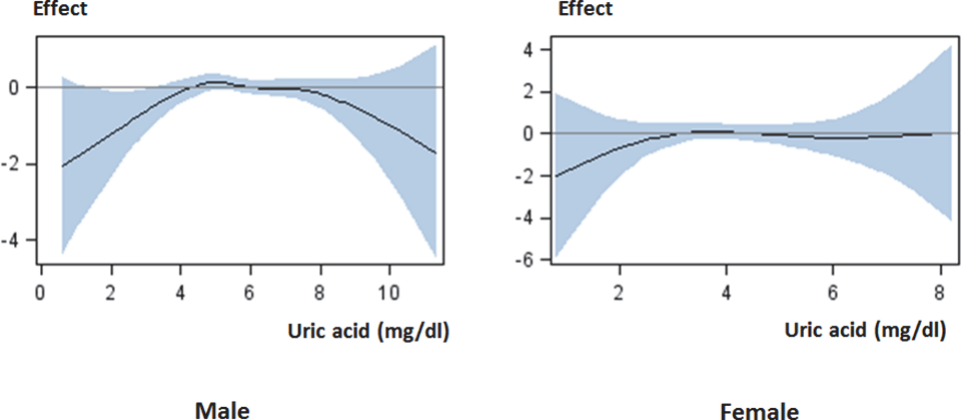
Effect of serum uric acid level on eGFR decline. The negative eGFR decline (rapidly decreasing eGFR) was observed in males with low or high serum uric acid levels, but not in females. The curves from generalized additive models with spline show the pattern of effect of serum uric acid level on eGFR decline with 95% confidence interval: male, linear *p* = 0.0001, spline *p* = 0.012; female, linear *p* = 0.074, spline *p* = 0.52. The models are adjusted for baseline characteristics. Abbreviations: Effect, effect of serum uric acid level on eGFR decline.

In both males and females, multivariate linear mixed models showed that eGFR decline was negatively associated with high serum uric acid levels for males, parameter estimates (standard error) -0.448 (0.0626), *p* = 0.0001; females -0.512 (0.159), *p* = 0.0014. Generalized additive models showed that both high and low serum uric acid levels contributed more to the decrease in eGFR than intermediate levels in males, but no relationships between serum uric acid level and eGFR decline were observed in females (Fig. 3).

The relationship between serum uric acid level and eGFR decline was examined in the subjects with low serum uric acid levels (group 1). Multivariate linear mixed models showed that serum uric acid level was positively associated with eGFR decline (Table 3).

**Table 3:** Association between high serum uric acid levels and eGFR decline in subjects with low serum uric acid levels (male, <5 mg/dl; female, <3.6 mg/dl).

	Male	Female
Uric acid	0.928 (0.234) *p* = 0.0001	1.346 (0.643) *p* = 0.045
Age	-0.188 (0.0173) *p* = 0.0001	-0.134 (0.0345) *p* = 0.0005
BMI	0.0731 (0.0596) *p* = 0.22	-0.0691 (0.121) *p* = 0.57
eGFR	-0.359 (0.0101) *p* = 0.0001	-0.360 (0.0156) *p* = 0.0001
Proteinuria	1.216 (1.396) *p* = 0.39	1.487 (2.023) *p* = 0.47
DM	-0.248 (0.853) *p* = 0.77	2.416 (2.485) *p* = 0.34
Hypertension	0.112 (0.708) *p* = 0.88	-3.833 (3.469) *p* = 0.28
Hyperuricemia	-2.879 (1.339) *p* = 0.034	
CVD	-1.495 (1.398) *p* = 0.29	1.702 (2.518) *p* = 0.50
Alcohol	0.385 (0.363) *p* = 0.29	0.244 (0.726) *p* = 0.74
Smoking	0.546 (0.349) *p* = 0.12	-1.437 (0.957) *p* = 0.14
Exercise	-1.081 (0.345) *p* = 0.0022	-0.665 (0.636) *p* = 0.30

Values are given as parameter estimates (standard error) and *p* values. Multivariate linear mixed models were adjusted for baseline characteristics. Age, BMI, serum uric acid level, eGFR, and proteinuria were treated as time-dependent variables in multivariate linear mixed models. Because there were only four females with hyperuricemia, hyperuricamia was not included in the model for females.

Abbreviations: BMI, body mass index; eGFR, estimated glomerular filtration rate; proteinuria, proteinuria positive; DM, diabetes mellitus; CVD, history of cardiovascular disease; hyperuricemia, history of hyperuricemia including gout; alcohol, daily alcohol consumption; exercise, having regular exercise.

### Exercise and Loss of Kidney Function

**Fig 4:**
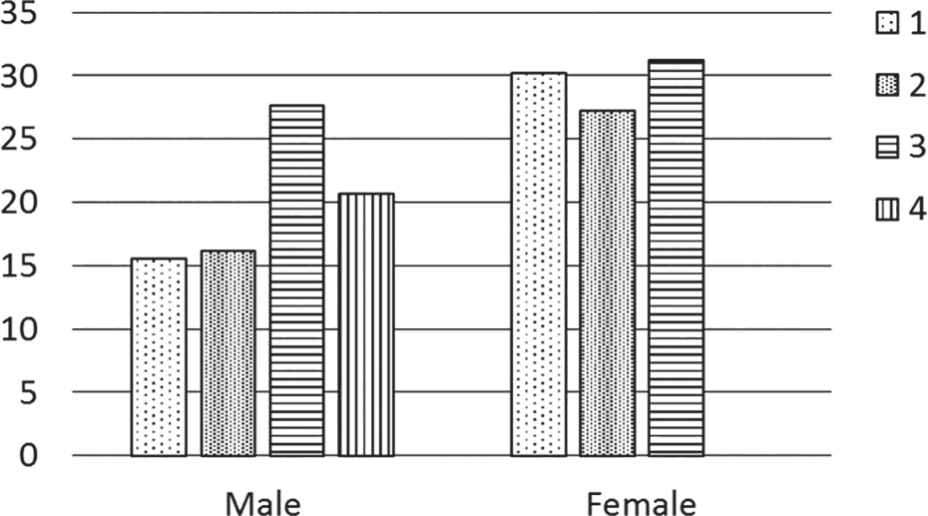
Exercise load and risk of loss of kidney function in subjects with low serum uric acid levels. The risk of loss of kidney function was increased by a high exercise load in males. Exercise load: Male; level 1, almost no exercise; level 2, once a moth; level 3, twice a month; level 4, once a week or more. Female; level 1, almost no exercise; level 2, once a moth; level 3, twice a month or more. Abbreviations: Incidence, incidence of loss of kidney function; Male, serum uric acid levels were less than 5 mg/dl; Female, serum uric acid levels were less than 3.6 mg/dl.

The effect of exercise on the loss of kidney function was examined in subjects with low serum uric acid levels. Regular exercise was negatively associated with the loss of kidney function in males (Table 3). A high exercise load tended to be associated with the outcome events in males (Chi-square test, *p* = 0.027), but not in females (*p* = 0.94) (Fig. 4).

## Discussion

In this study, a U-shaped association between the risk of loss of kidney function and serum uric acid level was demonstrated. This study showed that the risk of loss of kidney function was high for both males and females with high serum uric acid levels. The risk was also high for males with low serum uric acid levels. When subjects with low serum uric acid levels were focused on, eGFR decreased with low serum uric acid level for both males and females. Hyperuricemia has been reported as a risk factor for the development of CKD and the decrease in GFR, which is in agreement with our results [2–4]. However, the association between low serum uric acid levels and the loss of kidney function has not been shown in any large-scale studies; to the best of our knowledge, we are the first to report this association.

In this study, we showed that low serum uric acid levels were associated with the loss of kidney function. A report on a retrospective cohort study of health-check population in Taiwan has been the sole report that mentioned the above association thus far [15]. The results of the Taiwanese study suggest that low serum uric acid levels may increase the risk of CKD development (incident CKD), which however did not reach statistical significance. Researchers of the Taiwanese study used the subjects with serum uric acid levels of 2.0 to 4.5 mg/dl as the control group and calculated the hazard ratio for the subjects with serum uric acid levels lower than 2.0 mg/dl. Hypouricemic patients with serum uric acid levels lower than 2.0 mg/dl are rare [7]. In the Taiwanese study, the number of patients with hypouricemia was only 50 out of 94422 and the statistical power was weak, which may be the reason why no statistical significance was obtained. A Japanese study showed that patients with renal hypouricemia caused by the mutation of *SLC22A12* were among those with low but more than 2 mg/dl serum uric acid levels [16]. In our study, the range of low serum uric acid levels was wider than that in the Taiwanese study, which resulted in patients with mild hypouricemia to be included in the hypouricemic group. As a result, we found that low serum uric acid levels were associated with the loss of kidney function.

Our findings suggest that the upper limit of the range of low serum uric acid levels may be higher than 2 mg/dl. However, the exact upper limit is unclear. Although the outcome was different from that of our study, NHANES showed a U-shaped association between cardiovascular mortality and serum uric acid level [6]. The risk of cardiovascular mortality was high for males with serum uric acid levels lower than 5.0 mg/dl and for females with serum uric acid levels lower than 4.0 mg/dl. Suliman et al. reported a U-shaped association between mortality and low serum uric acid levels (lower than 5.3 mg/dl) in patients with CKD stage 5 [17]. According to a cohort study, cardiovascular morbidity was associated with low serum uric acid levels (lower than 3.9 mg/dl) [18]. In our study, low serum uric acid levels were defined as lower than 5 mg/dl for males and lower than 3.6 mg/dl for females, which were close to those reported in previous studies. From the above, it is considered necessary to regard people with low serum uric acid levels as being at high risks of cardiovascular morbidity and progression of loss of kidney function and to provide appropriate treatments.

The pathogenesis of loss of kidney function in patients with hypouricemia has not been completely clarified. The causes of hypouricemia have been categorized into (1) increased urinary excretion (renal hypouricemia) and (2) decreased production. The mechanisms underlying the loss of kidney function due to renal hypouricemia have been reported. A Japanese study showed that patients with renal hypouricemia (lower than 2 mg/dl) had experienced AKI with severe loin pain after anaerobic exercise (ALPE) [19]. According to that study, all the study patients later recovered their kidney function after developing AKI but 24% experienced recurrent AKI. Histopathological analysis revealed chronic lesions in some of the patients who experienced recurrent AKI despite a normal creatinine clearance rate. However, whether these patients developed CKD was unclear because the patients were not followed up. Regarding the pathogenesis of ALPE in patients with hypouricemia, the following is proposed: renal hypouricemia increases oxidative stress, which induces renal arterial spasm after exercise, resulting in the loss of kidney function [20]. A systematic review showed that AKI is a risk factor for CKD [21]. Thus, low serum uric acid levels may induce AKI recurrence resulting in CKD.

On the other hand, the mechanism underlying the loss of kidney function in patients with mild hypouricemia (male, 2 to 5.0 mg/dl; female, 2 to 3.6 mg/dl) has not been reported. Because patients with urinary hypouricemia tend to show very low serum uric acid levels (lower than 2 mg/dl), candidate causes in this range of serum uric acid levels may be the decreased production of uric acid caused by certain conditions, such as xanthinuria, purine nucleoside phosphorylase deficiency, xanthine oxidase inhibitors, and liver diseases. However, these conditions are very rare among healthy people. There may be other mechanisms leading to the loss of kidney function in patients with mild hypouricemia.

In this study, the mean serum uric acid levels were higher in males than in females. A similar tendency was reported in a cross-sectional study in Japan [22]. In this study, the risk of loss of kidney function was high for males with low serum uric acid levels. A cohort study in Japan showed that elevated serum uric acid levels were associated with cardiovascular mortality and that there were significant differences in the increased risk of cardiovascular death and serum uric acid levels between females and males [23]. From these results, it was suggested that serum uric acid levels may differently affect kidney function between males and females. The causes of the gender difference in the effects of serum uric acid levels on kidney function have not been fully clarified yet. Some of the possible causes are male and female hormones. It has been reported that estradiol, progesterone, and testosterone affect the uric acid transporters such as urate transporter 1 and glucose transporter 9, and sodium-coupled monocarboxylate transporter 1 in the mouse kidney [24,25]. In this study, it was also observed that exercise load was associated with the loss of kidney function in males with low serum uric acid levels. ALPE is more frequently observed in males than in females [26]. The difference in the prevalence of ALPE between male and females may underlie the difference in the effects of serum uric acid levels on kidney function.

A report showed that being male, daily exercise, and a lack of guidance from physicians were the risk factors for ALPE in patients with hypouricemia [19]. On the other hand, exercise has the benefits of preventing metabolic syndrome, diabetes mellitus, and cardiovascular diseases. A systematic review showed that exercise improves aerobic capability, muscular function, cardiovascular function, walking capacity and health-related quality of life [27]. ALPE in patients with hypouricemia is not caused by any type of exercise but caused mainly by short-term vigorous anaerobic exercise and dehydration [26]. Therefore, physicians should explain to males with low serum uric acid levels not only the benefits of aerobic exercise, but also the risk of ALPE, and instruct them to avoid vigorous anaerobic exercise and take sufficient amounts of water as preventive measures for ALPE.

Our study had several limitations. First, the subjects of this study were healthy people, and those with missing values were excluded from the study, which might have resulted in a selection bias. Second, the subjects who were not followed up were excluded from the analysis. Moreover, the accurate date of the onset of loss of kidney function was unclear because data obtained every three years were used. Therefore, the risks of low serum uric acid levels and the progression of loss of kidney function might have been underestimated. Third, details of any treatment provided to the subjects were not examined. Hence, the efficacies of antihyperuricemic medicines for hyperuricemia and renin-angiotensin-aldosterone system inhibitors in inhibiting the progression of CKD were not assessed. Fourth, the dietary habit of the subjects was not surveyed despite the fact that diet affects uric acid levels. Moreover, the type of exercise was not surveyed. Therefore, the effects of these factors on serum uric acid level were not assessed.

## Conclusions

In this study, there was a U-shaped association between the risk of the loss of kidney function and serum uric acid level. Low as well as high levels of uric acid were the risk factors for the loss of kidney function in healthy people. A further study is required by following up people with low serum uric acid levels in detail and clarifying the pathophysiological mechanism that links low serum uric acid levels to the loss of kidney function.
